# Hybridization Facilitates Adaptive Evolution in Two Major Fungal Pathogens

**DOI:** 10.3390/genes11010101

**Published:** 2020-01-16

**Authors:** Himeshi Samarasinghe, Man You, Thomas S. Jenkinson, Jianping Xu, Timothy Y. James

**Affiliations:** 1 Department of Biology, McMaster University, 1280 Main St. W, Hamilton, ON L8S 4K1, Canada; samaraya@mcmaster.ca (H.S.); youm1@mcmaster.ca (M.Y.);; 2 Department of Environmental Science, Policy, and Management, University of California, Berkeley, CA 94720, USA; tsjenkinson@berkeley.edu; 3 Department of Ecology and Evolutionary Biology, University of Michigan, Ann Arbor, MI 48109, USA

**Keywords:** *Cryptococcus*, AD hybrids, frog chytrid, reproductive incompatibilities, aneuploidy

## Abstract

Hybridization is increasingly recognized as an important force impacting adaptation and evolution in many lineages of fungi. During hybridization, divergent genomes and alleles are brought together into the same cell, potentiating adaptation by increasing genomic plasticity. Here, we review hybridization in fungi by focusing on two fungal pathogens of animals. Hybridization is common between the basidiomycete yeast species *Cryptococcus neoformans* × *Cryptococcus deneoformans*, and hybrid genotypes are frequently found in both environmental and clinical settings. The two species show 10–15% nucleotide divergence at the genome level, and their hybrids are highly heterozygous. Though largely sterile and unable to mate, these hybrids can propagate asexually and generate diverse genotypes by nondisjunction, aberrant meiosis, mitotic recombination, and gene conversion. Under stress conditions, the rate of such genetic changes can increase, leading to rapid adaptation. Conversely, in hybrids formed between lineages of the chytridiomycete frog pathogen *Batrachochytrium dendrobatidis* (*Bd*), the parental genotypes are considerably less diverged (0.2% divergent). *Bd* hybrids are formed from crosses between lineages that rarely undergo sex. A common theme in both species is that hybrids show genome plasticity via aneuploidy or loss of heterozygosity and leverage these mechanisms as a rapid way to generate genotypic/phenotypic diversity. Some hybrids show greater fitness and survival in both virulence and virulence-associated phenotypes than parental lineages under certain conditions. These studies showcase how experimentation in model species such as *Cryptococcus* can be a powerful tool in elucidating the genotypic and phenotypic consequences of hybridization.

## 1. Introduction

Hybridization refers to the interbreeding of individuals from genetically distinct populations or species. Through hybridization, genes and alleles that have diverged significantly from each other are brought together into the same cells and individuals, potentially creating the scenario of novel interactions among genes and genomes. There are two contrasting views on the roles of hybridization in organic evolution. On the one hand, hybridization is considered an evolutionary noise with limited long-term effects. Indeed, under a model in which species diverge by adaptation to different niches, the majority of hybrids are expected to show lower fitness than either parental genotype in parental niches [1,2]. On the other hand, certain hybrid progenies may display transgressive segregation/extreme phenotypes or fitness, both positive and negative, that exceed parental values, especially in novel ecological niches. Because of this, the contrasting view believes that hybridization plays critical roles in generating evolutionary novelty and can impact long-term evolution [3,4]. 

Hybrids may be either homoploid or polyploid. Homoploid hybrids have the same ploidy as the parents, but they may face obstacles to further sexual reproduction due to potential chromosomal incompatibilities. If homoploid hybrids can self-fertilize or backcross, the resulting offspring often reveals high genetic and phenotypic variance, including the generation of transgressive phenotypes [5]. Polyploid hybrids, specifically allopolyploids, have a higher ploidy generated typically by chromosome duplication, either in gametogenesis or after zygote formation following the mating of different species [6]. The presence of pairs of homologous chromosomes from each parental species alleviates the problems in meiosis due to low sequence similarity or structural rearrangements that lead to hybrid sterility in homoploids. Outcomes of hybridization are diverse. For example, in plants, hybridization may lead to the formation of novel hybrid species that are genetically isolated and phenotypically distinct from progenitors [3,5]. In other contexts, the extent of hybridization may be limited to a geographic region (or hybrid zone) maintained by either environment-genotype interactions or through a balance between migration and selection. In any case, hybridization also makes possible the introgression of alleles from one parental species into another, which is becoming easier to identify through genome sequencing [7,8].

In all major groups of eukaryotes, such as plants, animals, and fungi, natural hybridization has been reported, with some groups showing over 20% of extant species as hybrids. Previously, however, hybridization in fungi was either ignored or discounted, and only since the molecular revolution has hybridization in fungi been increasingly accepted as playing important roles [9,10]. Fungi are unique from the better-studied plants and animals in that they often produce copious (literally millions or billions) amounts of recombinants from a single mating event, which could lead to the generation of immense diversity. They are also unique because their dispersal is not considered particularly limited, such that traditional hybrid zones of a limited geographic extent seem unlikely. On the other hand, many fungi display a mixed mode of reproduction, with extensive generations of asexual reproduction interspersed with rare sexual reproduction [11]. Such versatility in reproduction could allow hybrids to propagate asexually for extended periods of time without suffering from a potential segregation load [12]. In fungi, F1 hybrids often display aneuploidy, diploidy, or higher ploidy, and because of the extra chromosome copies they can continue to diversify and adapt through mechanisms such as mitotic recombination and the gain/loss of individual chromosomes.

In this review, we explore these issues with a fungal view of hybridization described using two major fungal pathogens, the human-pathogenic *Cryptococcus* species and the amphibian chytrid fungus *Batrachochytrium dendrobatidis*. We argue that hybridization in these two groups of fungi reveals much about how speciation and hybridization in fungi is unique, and how hybridization will become an increasingly important concern for both the environment and human health in this era of rapid fungal evolution. The choice of these two species is based off of the authors’ experiences. However, the pair complement each other nicely. On the one hand, *Cryptococcus* (Basidiomycota) is a model system and easily manipulated in the lab, while *B. dendrobatidis* (Chytridiomycota), a species of great ecological significance, is far from a model system and lacks genetic tools. Insights into hybridization in these species require different approaches, but much has been revealed and facilitated by genomics.

## 2. Hybridization in *Cryptococcus* Species Complex

### 2.1. Cryptococcus Species Complex

**Figure 1:**
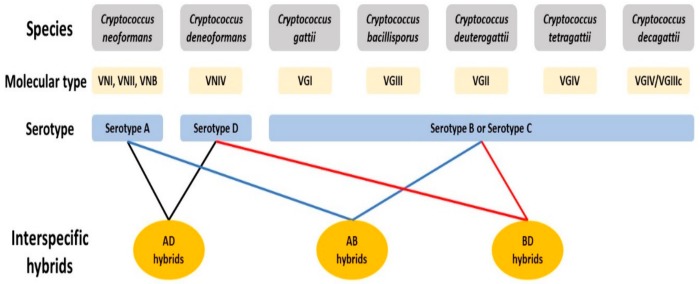
The currently recognized species in the pathogenic *Cryptococcus* species complex. The seven species of this complex can be distinguished based on genetic and molecular differences, and each is assigned a distinct molecular type based on clade assignment using multilocus sequence typing markers. Historically, cryptococcal strains were broadly categorized into serotypes based on the antigens found at the cell surface. Hybrids arising from mating between species are named based on the serotypes of the parental strains.

The human pathogenic *Cryptococcus* species complex comprises a group of closely related, basidiomycetous yeast species, responsible for over 220,000 annual, global infections with a mortality rate of ~80% [13]. Cryptococcal infections, known to occur in multiple forms including respiratory infections, skin lesions, and meningoencephalitis, are collectively called cryptococcosis and are a leading cause of death among HIV/AIDS patients worldwide. Commonly found in association with soil, bird droppings and tree barks, *Cryptococcus* species has a global distribution with strains having been discovered from all continents except Antarctica [14]. Currently, seven evolutionarily divergent lineages are recognized as pathogenic *Cryptococcus* species, which can be distinguished based on their genetic and molecular characterization, with each species having been assigned specific molecular types (Figure 1) [15,16]. Historically, *Cryptococcus* strains were categorized into serotypes based on the structure of polysaccharides at the cell surface. An alternative classification system aims to maintain two major species that are subdivided into varieties and/or molecular types [17,18]. In this review, we will use the seven-species classification system with (i) *C*. *neoformans* (serotype A, molecular types VNI, VNII and VNB; VN = variety *neoformans*), (ii) *C. deneoformans* (serotype D, molecular type VNIV), (iii) *C*. *gattii* (molecular type VGI; VG = variety *gattii*), (iv) *C. bacillisporus* (molecular type VGIII), (v) *C. deuterogattii* (molecular type VGII), (vi) *C. tetragattii* (molecular type VGIV) and (vii) *C. decagattii* (molecular type VGIV/VGIIIc) (the last five species share serotypes B and C). To maintain consistency with the previous literature, we will categorize cryptococcal hybrids based on the serotypes of the parental species. For example, hybrids of *C*. *neoformans* and *C*. *deneoformans* will be referred to as AD hybrids.

The seven species are believed to be descended from a common ancestor that existed on the supercontinent Pangea [19]. It is hypothesized that the ancestral population was split into two distinct groups following the breakup of Pangea and subsequent continental drift further spatially isolated the subgroups, leading to the emergence of two major, independently evolving populations in South America and Africa. This theory is supported by the fact that the estimated split of VN molecular types from VG molecular types occurred ~100 million years ago (mya) which coincides with the breakup of Pangea [19]. Furthermore, genomic analyses have revealed the origin of VN molecular types to be Africa, while VG molecular types originated in South America. The ancestral populations of VN and VG molecular types continued to diverge and evolve, likely due to local niche differences, eventually splitting into the seven species observed today. *C*. *neoformans* and *C*. *deneoformans* are believed to have diverged ~24.5 mya [19,20,21,22]. More recently, within C. *neoformans*, VNI and VNII split from each other ~4.7 mya. Among VG lineages, VGII diverged from other VG lineages ~12.5 mya, followed by VGIV splitting ~11.7 mya. VGI and VGIII were the last to diverge from each other, approximately 8.5 mya [21]. 

In the last century, historical spatial barriers between the seven cryptococcal species have been challenged with the significant increase in international commercial and human travel. The different lineages are now thrust back into contact due to anthropogenic transfer of cryptococcal cells/strains across countries and continents. Despite significant genomic nucleotide divergence, these species are still sufficiently compatible to initiate mating with each other, making interspecific hybridization a significant force that shapes their ongoing evolution. In fact, cryptococcal hybrids with superior fitness to parental strains have been recovered from both natural and laboratory settings while the proportion of infections caused by *C. neoformans* x *C. deneoformans* (AD) hybrids is on the rise, especially in Europe, where these hybrids are responsible for nearly 40% of all cryptococcal infections. The implications of interspecific hybridization on the adaptive evolution of the pathogenic *Cryptococcus* species complex are discussed below.

### 2.2. Sexual Cycle of Cryptococcus 

**Figure 2:**
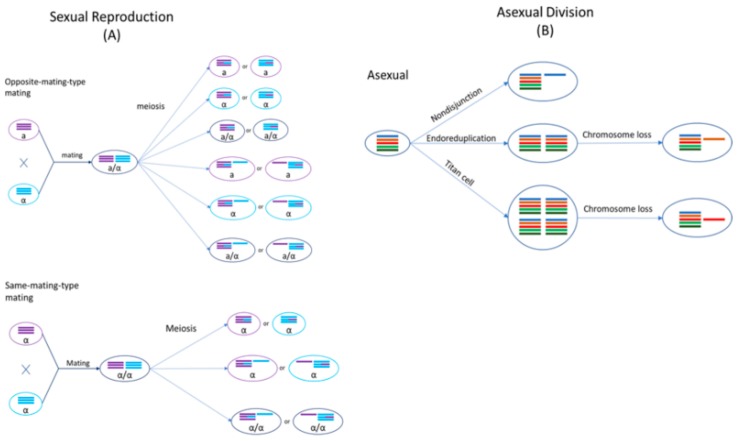
Genomic changes of *Cryptococcus* occur by sexual and asexual processes. (**A**) In sexual mating and subsequent meiosis, resulting in the formation of recombinant cells, both euploidy and aneuploidy can be observed; (**B**) Asexual replication can undergo nondisjunction, endoreduplication, or the formation of titan cells to cause genomic changes.

*Cryptococcus* species are haploid basidiomycete yeasts that can reproduce asexually via budding or sexually via mating (Figure 2). The sexual cycle of *Cryptococcus* was first observed by Kwon-Chung four decades ago [23,24]. Under nutrient-limiting conditions (e.g., low nitrogen) and dehydration, cells of opposite mating types (*MAT***a** and *MAT***α**) could be triggered to fuse and form a zygote. A germ tube originating from the *MAT**a*** end of the zygote extends out and subsequently develops into dikaryotic hyphae with the two parental nuclei maintained as separate entities. During hyphal growth, a specialized hypha called a clamp connection forms across septa and fuses with the subapical neighboring cell. One of the two daughter nuclei in the apical cell is transferred to the subapical cell via the clamp connection to reform the dikaryon. Haploid blastospores can form along the hyphae: blastospores are vegetative, yeast-like cells that bud from the hyphae. Some hyphal cells can also enlarge and form chlamydospores, which are thick-walled vegetative cells with a condensed cytoplasm. Chlamydospores can facilitate the long-term survival of cells in harsh environments [25]. At the onset of meiosis, the tip of an aerial hypha enlarges to form a basidium within which the two parental nuclei fuse and meiosis occurs to produce four recombinant, haploid nuclei. The daughter nuclei undergo repeated mitotic divisions with individual nuclei packaged into individual basidiospores that bud off from the basidium, consequently forming four chains of basidiospores [26,27,28]. 

Sexual mating in *Cryptococcus* is normally restricted to cells of opposite mating types. However, cryptococcal cells of the same mating type could be induced to mate with each other under nutrient-limiting conditions, referred to as same-mating-type reproduction [29,30,31]. Mating between genetically different strains of the same mating type can produce a high frequency of recombinants, similar to opposite-mating-type mating [27]. Even though both *MAT***a** and *MAT***α** cells can undergo same-mating-type reproduction, the presence of cells with opposite mating types significantly enhances this process [32]. *MAT***α** is the predominant mating type in natural populations, with >99% of *C*. *neoformans* strains and ~95% of other cryptococcal strains belonging to this mating type [14,33,34]. During same-mating-type mating, haploid *MAT***α** cells become **α**/**α** diploids, either by endoduplication or by nuclear fusion following cell–cell fusion between two **α** cells [27,35]. The newly formed diploid nucleus then undergoes one round of meiosis to produce four, recombinant haploid nuclei, which are then packaged into basidiospores [36]. Evidence for same-mating-type mating in *Cryptococcus* has also been found in nature: particularly, same-mating-type reproduction could be beneficial due to natural populations in both clinical and environmental settings being predominantly of the same mating type, *MAT***α** [37,38]. 

Both mating processes confer two major benefits to *Cryptococcus*: (i) they result in the production of basidiospores which act as the unit of long-distance dispersal, and (ii) they are known to be mutagenic, generating both genetic and phenotypic variation, as described in the sections below [39]. Strains of different cryptococcal species have been successfully mated with each other in the laboratory, albeit generally with low spore germination rates [40]. Unlike haploid parental strains, the resulting interspecific hybrids are often diploid or aneuploid, indicating that genomic divergence between parental lineages could hinder chromosomal disjunction and proper meiosis during gamete formation.

### 2.3. Hybrids in the Cryptococcus Species Complex

Cryptococcal hybrids, discussed below, are categorized according to the serotypes of the parental lineages.

AB and BD hybrids: To the best of our knowledge, hybrids arising from the mating of *C. neoformans* (serotype A) or *C. deneoformans* (serotype D) with one of *C. gattii, C. bacillisporus, C. deuterogattii, C. tetragattii* or *C. decagattii* (serotypes B or C) have not been recovered from the environment. However, there have been several reports of infections caused by AB (serotype A x serotype B) and BD (serotype B x serotype D) hybrid strains. The first such hybrid was reported in 2006, when three strains isolated from the cerebrospinal fluid of two patients in the Netherlands were determined to be diploid strains of serotype BD [41]. Based on these findings, it was estimated that BD hybrids made up about 1% of the clinical isolates found in the Netherlands between 1977 and 2005. A cryptococcal hybrid of serotype A x serotype B was first reported in 2008, when a strain isolated from an AIDS patient in Canada was determined to be an AB hybrid [42]. In 2012, four AB hybrid strains were identified among clinical samples obtained from the cerebrospinal fluid of patients in Brazil (*n* = 2), Columbia (*n* = 1) and India (*n* = 1) [43]. AB and BD hybrids have since been reported in Germany [44], Denmark [45], and the United States [46], all from clinical sources. Even though mating between the parental lineages can be induced in the laboratory, the mating cells often fail to complete the sexual cycle, indicating a strong post-zygotic reproductive barrier [24]. These observations suggest that, while these species are still genetically and phenotypically compatible enough to initiate mating, they produce relatively few viable hybrid progenies, leading to their rare occurrence in environmental and clinical samples. Furthermore, to date, cryptococcal hybrids of serotypes AC and CD have never been reported in the literature.

AD hybrids: Hybridization between A and D serotypes is a significantly more common occurrence than the hybridization of A or D serotypes with B or C serotypes. Indeed, *C. neoformans* x *C. deneoformans* hybrids, commonly referred to as AD hybrids, are the most common of all cryptococcal hybrids. AD hybrids are assigned their own molecular type of VNIII. Since their initial discovery in 1977, their prevalence has been steadily increasing, with AD hybrids currently causing up to 40% of all cryptococcal infections in Europe [47,48,49,50]. They have been recovered from clinical and environmental sources in many countries, across most continents [51]. AD hybrids are typically diploid or aneuploid and experience frequent loss of heterozygosity during vegetative growth [52,53,54].

BC hybrids: Under mating-inducing conditions, strains of different VG molecular types (serotypes B and C) are capable of mating with each other in the laboratory [40,55,56,57]. However, to the best of our knowledge, diploid/aneuploid VG hybrid strains (BC hybrids) have not been recovered from environmental or clinical sources. One likely explanation might be that VG lineages are still sufficiently compatible with each other to produce haploid, recombinant progeny: nucleotide divergence between VG lineages is less extensive in comparison to that between VNI and VNIV (see Section 2.1). In fact, when strains of serotypes B and C are mated with each other, they produced proper dikaryotic hyphae with clamp connections that were morphologically similar to those produced when VG strains of the same serotype are mated with each other [24,55]. However, spore viability was found to be very low (<1%) in lab-derived reciprocal crosses of VGII (*MAT***α**) × VGIII (*MAT***a**) and VGII (*MAT***a**) x VGIII (*MAT***α**): among the spores that successfully germinated, 18/18 and 9/16 were diploid/aneuploid in the two crosses, respectively [58]. Furthermore, almost all collected F1 hybrid progeny were determined to be diploid/aneuploid when VG lineages were mated with each other, or VGIII was mated with VN lineages in the laboratory (You et al., unpublished data). Together, these observations suggest that VG hybrids can be produced, and the apparent absence of diploid/aneuploid VG hybrids in nature is likely due to these hybrids’ failure to successfully compete with parental lineages in their natural habitats. 

## 3. Outcomes of Hybridization between Cryptococcal Lineages 

### 3.1. Hybrid Inviability

Spore germination rates in hybrid cryptococcal crosses are typically low, indicating significant post-zygotic reproductive isolation between these species. Studies have reported ~5–20% of hybrid spores to be viable in lab-derived hybrid crosses between *C*. *neoformans* and *C*. *deneoformans* [52,59,60]. However, the true germination rate might be slightly higher, since we have observed some AD hybrid spores to display an abortive phenotype where, following germination, growth is aborted after several mitotic divisions, indicating genetic incompatibilities within the nucleus [61]. Two recent studies detected significant variability in spore germination rates in crosses between strains of *C. gattii, C. bacillisporus, C. deuterogattii, C. tetragattii and C. decagattii,* with a range of ~1–98% [29,40,60]: however, since the ploidy of germinated spores was not determined in the study by You et al., the proportion of viable progeny that were diploid/aneuploid is not known. You et al. also found spore viability to vary between ~1–43% in a series of crosses where a *Cryptococcus bacillisporus* strain was crossed with different *C*. *neoformans* and *C. deneoformans* strains [29]. The significant variation in spore viability observed across multiple studies highlights the complex interplay of determinants, including parental genetic backgrounds as well as environmental and genotype–environment interaction effects, on the germination of hybrid cryptococcal spores [29,58,59].

### 3.2. Hybrid Sterility 

While most cryptococcal hybrids are heterozygous at multiple loci across the genome and contain alleles for both mating types (i.e., heterozygous at the *MAT* locus), few are self-fertile or can mate with other strains [52,61]. Basidiospores produced by three self-fertile AD hybrids containing both mating types germinated at a very low rate of ~5% in laboratory conditions: these three AD hybrids did not produce any sexual spores when co-incubated with haploid *MAT***a** and *MAT***α** strains of *C*. *neoformans* and *C. deneoformans* [52]. However, sterility does not pose a barrier to cryptococcal hybrid success, as they can propagate asexually via mitosis.

### 3.3. Phenotypic Diversity and Hybrid Vigor

Studies investigating the phenotypes and virulence of cryptococcal hybrids have found variable results with some reporting hybrid vigor (heterosis) while others have found hybrids to be inferior to parental strains. In general, AD hybrids are less virulent than either *C. neoformans* or *C. deneoformans* haploid strains, though this result is not always observed [62]. However, increasing evidence of hybrid vigor in both natural and laboratory-constructed AD hybrids has been found. For example, AD hybrids might be better able to adapt to new environmental niches than *C*. *neoformans* and *C. deneoformans* isolates. Natural AD hybrid strains are more resistant to UV irradiation than native *C*. *neoformans* strains from Botswana [63]. Laboratory-constructed AD hybrids also showed hybrid vigor with higher resistance to both UV irradiation and high temperatures than *C*. *neoformans* and *C. deneoformans* parents [63,64]. Another study found the majority of 31 investigated global AD isolates to be resistant to the antifungal drug FK506 [54,65]. A small proportion of hybrid strains in an AD hybrid population derived from a cross between CDC15 (*C*. *neoformans*, MAT**α**) and JEC20 (*C*. *deneoformans*, *MAT***a**) was found to surpass both parents in the expression of essential virulence factors, including melanin production, capsule production, growth at 37 °C, resistance to the antifungal fluconazole, and cell size [66]. The remaining hybrid offspring displayed intermediate phenotypes or inferior phenotypes to both parents. At present, similar phenotypic data on other cryptococcal hybrids are not available due to their rarity compared to AD hybrids. However, the presence of these hybrids in clinical settings suggests that at least some are capable of causing fatal infections in humans. 

The phenotypic diversity found among cryptococcal hybrids indicates the presence of extensive genetic diversity in hybrid populations. Due to significant genomic differences between the parental species, frequent chromosome nondisjunction is observed during meiosis, with hybrids often inheriting novel and/or unique combinations of chromosomes. For example, evidence of homozygosity (or hemizygosity) interspersed with heterozygosity is observed across AD hybrid genomes [59,66,67]. Homozygosity could be derived through either chromosome loss or mitotic gene conversion, leading to loss of heterozygosity [54,65,68]. The generation of novel allelic and chromosomal combinations can offer cryptococcal hybrids a significant advantage in adapting to a diversity of environmental niches and competing with parental lineages for resources.

## 4. Genetics of Cryptococcal Hybrids

### 4.1. Aneuploidy in Cryptococcal Hybrids

AD hybrids are either diploid or aneuploid, as determined by fluorescence-activated cell sorting (FACS) analysis [49,52]. Previous studies revealed that aneuploidy in AD hybrids is most likely caused by the non-disjunction of homologous chromosomes during meiosis due to nucleotide sequence divergence (10–15%), as well as genetic incompatibilities, between *C. neoformans* and *C*. *deneoformans* genomes [52,69,70]. Sun and Xu found that at least one out of 114 screened co-dominant loci was heterozygous in the majority of lab-derived AD hybrid offspring strains, with an average heterozygosity of ~75% per strain [53]. Recombination between markers located on the same chromosome was observed, confirming the involvement of a meiotic process in the generation of these progeny, although the rate of crossovers was significantly lower during hybridization than that observed in intraspecific crosses of *C. neoformans* and *C. deneoformans*. Another analysis of a hybrid cross between H99 (*C*. *neoformans*, *MAT***α**) and JEC20 (*C*. *deneoformans*, *MAT***a**) suggested that the resulting hybrid progeny were likely generated via random nuclear fusion of two of the four recombinant nuclei generated from meiosis, which could result in heterozygous hybrids with doubled ploidy levels [71]. 

Clinical BD hybrid strains have also been found to be diploid or aneuploid by Bovers et al. [42]. These hybrids have a unique Amplified Fragment Length Polymorphism (AFLP) genotype (AFLP genotype group 8), revealing that they likely originated from hybridization between a *MAT***α**, serotype B strain (AFLP genotype 4) and a *MAT***a,** serotype D strain (AFLP genotype 2). Interestingly, these hybrids were heterozygous at two of the genotyped loci, namely RNA polymerase II (RPB2) and laccase (LAC): however, they were homozygous for the serotype B parent’s genotype at the Internal Transcribed Spacer (ITS) regions of the nuclear ribosomal RNA gene cluster. In addition, most BD hybrids were homozygous for the serotype B allele at the Intergenic Spacer (IGS) sequence of the nuclear ribosomal RNA gene cluster, while the remaining hybrids were homozygous for the serotype D allele.

While diploid/aneuploid hybrid strains of *C. gattii, C. bacillisporus, C. deuterogattii, C. tetragattii* and *C. decagattii* have not been recovered from nature, lab-derived hybrids of such crosses often display diploidy or aneuploidy. In two laboratory crosses between *C. bacillisporus* and *C. deuterogattii*, 18/18 and 9/16 of the spores that successfully germinated were determined to be diploid/aneuploid, respectively [58]. Furthermore, almost all collected F1 hybrid progeny were determined to be diploid/aneuploid when these five species were mated with each other, or when *C. bacillisporus* was mated with C. *neoformans* and *C. deneoformans* in the laboratory (You et al., unpublished data). 

### 4.2. Loss of Heterozygosity

The definition of loss of heterozygosity (LOH) is the loss of one parental allele in a certain genomic region in a heterozygous individual. LOH can be caused by multiple mechanisms, such as unbalanced chromosome rearrangements, gene conversion, mitotic recombination, and loss of a chromosome or a chromosomal segment. Double-strand break repair can give rise to short-range LOH events by gene conversion without crossover. In contrast, long-range LOH events are mostly caused by single crossovers or break-induced replication [72]. In addition, whole-chromosome LOH can arise from chromosome loss through nondisjunction, followed by duplication of the remaining homolog [73]. The duplication of a chromosome is a common occurrence in whole-chromosome LOH. With complete duplication of the remaining genetic material, the appearance of a normal karyotype is maintained, even though there may have been a wholesale loss of genetic diversity. Generally, LOH is not reversible, however, cells can regain the lost heterozygous alleles via outcrossing or mutation.

The emergence of LOH is considered a major mechanism of generating genetic diversity in populations of diploid heterozygous organisms. Unlike the parental haploid lineages, cryptococcal AD hybrids are often highly heterozygous, and may be prone to LOH, both during hybridization events and during asexual growth following germination [39,49,52,54,64]. During sexual mating, the two parental nuclei have been observed to fuse at earlier stages of sexual development (e.g., in the zygote or hyphae), providing opportunities for mitotic recombination to facilitate LOH at certain chromosomal regions before meiosis. A recent analysis of 297 lab-derived AD hybrid progeny strains generated from a single cross revealed the hybrids to experience extensive loss of chromosomes [74]. Both partial and complete chromosome loss and duplication have been observed in some AD hybrids. Partial chromosome loss may result in genome rearrangement or the formation of novel chromosomes through truncation or translocation [54]. Li et al. found that the progeny strain P5 (progeny of a self-fertile AD strain CDC228) partially lost some chromosomes (Chromosomes 8 and 10 from the C. *neoformans* parent) and completely lost some others (Chromosomes 5 and 13 from *C*. *neoformans*, and Chromosomes 3 and 12 from *C. deneoformans*), giving rise to a highly unique genome organization [54]. 

Interestingly, natural AD hybrids show a preferential retention of specific alleles and chromosomes from one of the two parents, suggesting that those alleles may offer survival and growth benefits under specific conditions [68]. Allele distributions in the genomes of AD hybrids often show significant departures from Mendelian ratios with alleles of one parent preferred over that of the other at certain loci [74]. In fact, Samarasinghe et al. [75] found genome-wide allele distribution in 297 AD hybrids to be significantly skewed in favor of the *C. deneoformans* parent from which the hybrids inherited mitochondria. It is hypothesized that given the uniparental mitochondrial inheritance seen in cryptococcal species, hybrids prefer to retain chromosomes of the mitochondria-donor parent to minimize incongruence between their mitochondrial and nuclear genomes. 

A very recent study conducted by Dong et al. estimated the rate of LOH during mutation accumulation in a laboratory-constructed diploid AD hybrid (CDC15 × JEC20) during mitotic divisions. They used 33 genetic markers located on 14 chromosomes to determine genome-wide allele distributions in the AD hybrid [76]. The parental haploid strain CDC15 (serotype A, *MAT***α**) is more resistant to fluconazole (minimum inhibitory concentration [MIC] = 64 µg/mL) than the other parent JEC20 (serotype D, *MAT***a**, MIC = 4 µg/mL). Their findings showed that only a few LOH events occurred over 800 generations of propagation on nutrient-rich medium, with an estimated rate of 6.44 × 10^−5^ LOH events per mitotic division. However, fluconazole exposure resulted in a dramatic 50-fold increase in LOH rate at two markers on Chromosome 1. Interestingly, Chromosome 1 contains two genes, *ERG11* (the fluconazole target gene) and *AFR1* (a major transporter for triazoles), both of which play major roles in the development of antifungal resistance in *C. neoformans* [66,75]. Here, the AD hybrid lost the fluconazole-susceptible allele of both genes inherited from JEC20 while maintaining the alleles from CDC15. In these evolved strains, the copy number of Chromosome 1 inherited from CDC15 also increased. Results from this study suggested that hybridization can facilitate the rapid adaptation of *Cryptococcus* to stressful environmental conditions. 

### 4.3. Dynamic Ploidy Changes in Cryptococcus 

Ploidy change is often associated with sexual reproduction. Fungal cells generally mate with cells of identical ploidy levels, resulting in intermediate sexual structures with double the genomic content. Subsequent meiosis reduces the DNA content by half, reinstating the original ploidy of the parental strains. Some fungi, like *Saccharomyces cerevisiae*, favor propagation in the diploid state while other fungi, like *Schizosaccharomyces pombe*, prefer to propagate in the haploid state with a transient diploid state, as is the case observed in *Cryptococcus* [77,78,79].

Cryptococcal cells isolated from clinical and environmental settings are normally haploid with 14 chromosomes. FACS analyses have revealed an appreciable proportion of AD hybrids to be diploid [46,52,80]. However, it is possible that the diploid AD hybrids are not heterozygous at all loci across the genome: the remaining copy of a chromosome is often duplicated following LOH events, maintaining diploidy. Environmental stress has been observed to induce chromosome mis-segregation causing chromosomal instability. For example, exposure to a high-dose fluconazole treatment can result in the amplification of Chromosome 1 in both haploid C. *neoformans* and AD hybrids [77,78]. A comparison of haploid and diploid *C. neoformans* cells found that haploid cells were generally more virulent than diploid cells in a murine inhalation model of cryptococcosis [64]. However, in a rabbit infection model, diploids displayed similar virulence levels to haploid forms [79]. Higher ploidy was found to be associated with larger cell size in C. *neoformans*. For example, titan cells that can grow up to an impressive 100 µm in diameter contain 16, 32, 64 or more copies of the genome [81]. The large size facilitates the survival of titan cells during infection by hindering ingestion by host macrophages and by imparting resistance to oxidative and nitrosative stresses [73]. However, in certain cases, increased ploidy has been shown to have modest detrimental effects on virulence in a murine inhalation model, growth at high temperature, and melanisation [64]. In addition, melanin production was found to be correlated with monosomy at Chromosome 13, while disomic variants produced less melanin and were less virulent in mice in *C. neoformans* cells isolated from AIDS patients [82]. The plasticity of their genomes provides cryptococcal hybrids with the flexibility to alter their ploidy, via chromosome loss/gain or duplication, which in turn promotes adaptation to a wide range of environmental conditions.

### 4.4. Cryptococcus as a Model System for Fungal Hybridization

Hybrids face significant challenges to survival and functionality due to two divergent genomes residing in the same cell. In a process referred to as genome stabilization, hybrids eliminate unfavorable combinations of the two parental genomes via a variety of mechanisms including recombination, gene conversion and chromosome loss [76]. The rate at which genome stabilization is achieved in a hybrid may be related to the extent of divergence between the two parental genomes, since incompatibilities between more differentiated genomes will be resolved faster within the hybrids. For example, aneuploidy is commonly found in hybrids derived from two divergent parents, while frequent LOH events can be viewed as a mechanism of achieving genome stabilization. 

Chromosomal nondisjunction during meiosis coupled with LOH during vegetative growth leads to the creation of cryptococcal hybrids with novel and unique allelic combinations not found in parental species. The novelty and plasticity of their genomes have put cryptococcal hybrids at a unique position to dynamically adapt to novel environmental niches and compete with parental lineages in current habitats. In fact, hybrid vigor, displayed by some hybrids in laboratory settings, and the increasing presence of AD hybrids in clinical samples suggest an advantage of cryptococcal hybrids to successfully adapt to the changing environment. In summary, genomic plasticity likely facilitates the rapid adaptation of hybrids to new environmental niches (e.g., harsh environments) or genetic perturbations. 

In a world where frequent international commerce and human travel is blurring geographical and spatial boundaries, hybridization between closely related taxa is an increasingly likely outcome across all kingdoms of life. The seven species of the *Cryptococcus* species complex provide an excellent model system for studying hybridization in fungi. Many tools have been developed and optimized for the study of these yeasts, including transformation [83], ploidy estimation by flow cytometry [84], and a strain-typing system integrating numerous well-characterized strains [85]. Importantly, there are multiple host models of cryptococcal infections that make in vivo experiments feasible [86]. Furthermore, whole genome sequences of hundreds of isolates from various geographical origins are available on online databases (e.g., NCBI, FungiDB) while gene editing in these species can now be carried out with high efficiency using specially adapted CRISPR-Cas9 [87]. Finally, experimenting on haploid yeasts such as *Cryptococcus* is more convenient, and the findings can be more generalizable to understanding pathogenicity as compared to the well-established model *Saccharomyces cerevisiae*, which is primarily diploid in nature and the lab. Insights gained from cryptococcal hybrid research can be used to guide research strategies on hybridization in lesser known pathogenic fungi such as *B. dendrobatidis*, discussed in the remaining sections of this review. The major obstacles in understanding the process of hybridization in non-model species are typically the lack of genetic tools, such as ability to conduct gene disruption, the inability to perform genetic crosses, or both. Fortunately, much can be understood about the process of hybridization using genomic characterization of wild collected strains. The genomic tools developed in model systems can be adapted to study the non-model fungal hybrids.

## 5. Hybridization in an Aquatic Chytrid Fungus Associated with Amphibian Declines

### 5.1. The Amphibian Chytrid Batrachochytrium Dendrobatidis

The chytrid fungus *Batrachochytrium dendrobatidis* (*Bd*) is a broad host-range pathogen that is known to infect close to 700 species of frogs, salamanders, and caecilians worldwide [88]. *Bd* is now recognized as the major contributor to near-simultaneous amphibian population declines in the 1980s and 1990s that are correlated with arrival of the pathogen [89,90]. To date, *Bd* has been detected on every continent except Antarctica [91] and is most likely introduced from source populations in east Asia [92].

*Bd* is composed of at least four deeply divergent evolutionary lineages with varying geographical histories and virulence against hosts. The most widespread and well documented of these lineages is a globally-distributed, hypervirulent diploid genotype, BdGPL (Global Panzootic Lineage) [93]. The other, putatively less virulent lineages of *Bd* include: a Brazilian lineage endemic to the Atlantic Forest region of southern Brazil, BdBrazil (also known as BdBrazil/Asia2) [92,94]; an African lineage endemic to the Cape region of South Africa, BdCape [93]; and an endemic Asian lineage BdAsia1, believed to be closest to the source of origin for Bd diversity [92]. Recently, the existence of an additional endemic lineage, BdAsia3, widespread throughout southeast Asia was reported from amphibian swabs [95]. 

### 5.2. Hybrids in Batrachochytrium Dendrobatidis

Intraspecific hybrid strains resulting from outcrossing between parental genotypes of divergent lineages are rare, but known to occur. Within the divergent lineages of *Bd*, reproduction appears to be strictly asexual [96], with the exception of BdAsia1, which has a population signature of a highly recombining population [93]. Both the paucity of outcrossing in natural *Bd* populations and the inability to cross isolates is a major point of contrast between *Bd* (and other non-model species) and the model fungal species with hybrids, such as *Cryptococcus*. Outcrossing among the divergent *Bd* lineages (referred to here as hybridization) is only known to occur in secondary contact zones where divergent lineages have been brought into proximity by human activity. There are currently five hybrid *Bd* isolates reported in the literature from two hybrid zones. Three of these hybrid isolates were documented from the Atlantic Forest of Brazil within a narrowly restricted zone in the southern Brazilian state of Paraná [94,97,98]. This locality is one of the areas where the BdGPL and BdBrazil lineages overlap. The other two known *Bd* hybrids are described from the Eastern Cape Province of South Africa where BdGPL and BdCape overlap. Putative hybrid isolates were also identified from genotyping DNA from amphibian skin swabs, but these could also be explained by coinfection [95]. Unlike the narrow geographic range of the Brazilian hybrid zone, the two South African hybrid isolates were collected approximately 200 km apart from one another [92]. Additional regions where secondary contact could occur along with hybridization are Europe, western Africa, and Central America [92,95,98]. These regions are of interest with respect to hybridization because they harbor BdGPL and BdCape lineages which are already known to hybridize. Evidence of hybridization derives from combination of otherwise lineage-specific alleles into the same genome, increased heterozygosity, and Bayesian admixture analyses [92,97,98]. Most of the hybrids appear to be F1, and an earlier F2 reported [94] was later determined to be an F1 which had undergone some LOH [97]. In most *Cryptococcus* hybrids, divergence in the chromosomal structure greatly hinders the ability to undergo meiosis. As a result, in *Cryptococcus*, most natural hybrids appear to be close to F1, and these data also agree with expectations of chromosomal pairing problems reflected in the 100 million year divergence between C. *neoformans* and *C. deneoformans* [19,20,21,22] and the studies which show meiotic segregation in F1 hybrids to be highly abnormal [53]. On the other hand, for *Bd*, there is no evidence that F1 hybrids have higher ploidy than the parental genotypes and, though the timeframe of divergence between the lineages of *Bd* is debated [92,97], the extreme end of an estimate of 100,000 years of divergence before hybridization is three orders of magnitude younger than cryptococcal hybrids. These data would predict that hybrids should be fertile. 

**Figure 3:**
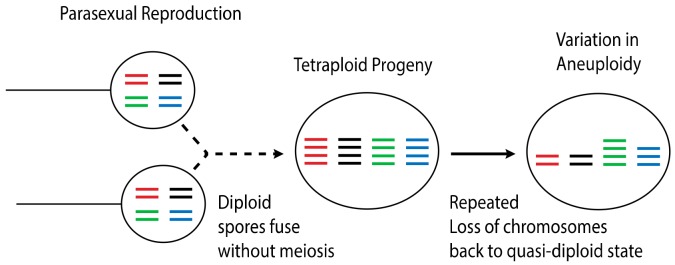
Parasexuality is a process of reproducing without a reductive cell division (meiosis). With parasexuality, a tetraploid offspring is produced, which is a transient stage followed by random loss of chromosomes during vegetative growth.

While clear genetic evidence of hybridization in *Bd* exists, mating and hybridization have not yet been observed in situ or in the laboratory. Likewise, specialized meiotic structures have never been reported for this species. The cellular process of hybridization in *Bd* is of special interest to understanding the dispersal ecology of this pathogen, because sexual reproduction in related members of the Chytridiomycota results in the production of harsh environment-resistant resting spores which may facilitate environmental or long-range transmission. Alternative cellular mechanisms of outcrossing that do not involve meiosis have also been proposed. One alternative mechanism by which *Bd* may be outcrossing is through a parasexual cycle [98]. Parasexual reproduction is well documented in other groups of pathogenic fungi, such as *Candida albicans* [99,100]. This mode of reproduction involves the fusion of diploid cells without meiosis. The resulting cell is a tetraploid intermediate which, in most cases, loses chromosomal copies back to a diploid state (Figure 3). Such a reproductive mechanism may explain the varying levels of aneuploidy prevalent throughout individual *Bd* isolates, as well as the lack of obvious meiotic structures or resting spores in this species. On the other hand, given the diversity of mechanisms possible to create aneuploidy, as discussed above for *Cryptococcus*, it is plausible that the sexual cycle of *Bd* is typical for other fungi, cryptic as it may be.

### 5.3. Outcomes of Hybridization in Batrachochytrium Dendrobatidis

The phenotypic outcomes of hybridization in *Bd* remain largely unknown. It is unclear whether hybrids are favored by natural selection in the habitats in which they were created. In order to test this, it would be useful to return to regions where hybridization has occurred in order to attempt reisolation of the same hybrid genotypes and to estimate the frequencies of hybrids related to parental species. Both measures can test whether hybrid genotypes are on the increase, which is predicted if they are favored by selection. In the only currently available study on hybrid phenotypes, Greenspan et al. [101] showed that hybrid virulence and pathogenicity was highly dependent on the infected host species. In a virulence challenge assay, the authors infected two endemic Brazilian, direct-developing frog species with BdGPL, BdBrazil, or hybrid isolates produced by the two lineages. In one host species, the high-altitude endemic pumpkin toadlet (*Brachycephalus ephippium*), hybrid isolates were more virulent (causing greater mortality in host animals) than either BdGPL or BdBrazil isolates. The endemic BdBrazil was the least virulent in this host species. In the other host species tested, the robber frog (*Ischnocnema parva*), hybrid isolates displayed an intermediate degree of virulence, with BdGPL being the most and BdBrazil being the least virulent.

Hybrid pathogenicity also varied according to host context. In the Greenspan et al. [101] study, the authors examined pathogenicity among isolates in three host species by assessing pathogen load upon host mortality. Again, the pathogenicity phenotypes of hybrids depended on the host species. In the host species, *B. ephippium*, where hybrid isolates were most virulent, the hybrids were also more pathogenic, with hybrid strains producing the highest spore loads on hosts at the time of mortality. In *I. parva*, the host species in which hybrid virulence was intermediate between BdGPL and BdBrazil, spore loads produced by hybrid isolates were comparable to those of BdGPL. The third species examined in the Greenspan et al. [101] study, the habitat-generalist, swamp treefrog (*Dendropsophus minutus*), is known to be more tolerant to *Bd* infection in laboratory challenge assays [102]. Because of this, infection experiments did not produce sufficient mortality in this species to analyze differences in virulence. However, the authors showed that *Bd* pathogenicity varied by genotype in this host species. Hybrid isolates produced spore loads intermediate between BdGPL (highest loads) and BdBrazil (lowest) when the host animals were assessed 60 days post-inoculation.

In summary, much work remains to be completed in understanding the cellular processes and phenotypic outcomes of hybridization in *Bd*. However, the Greenspan et al. study documents two interesting results. First, hybrid genotypes do show some degree of heterosis (hybrid vigor) in both virulence and pathogenicity characteristics. Second, and very importantly, this increase in the virulence and pathogenicity of the hybrid strains are context-dependent on the host species being infected. These results highlight that predicting the phenotypic characteristics of hybrid *Bd* genotypes is very complex, and further experimental work should focus on assessing hybrid characteristics in a broad range of host species.

### 5.4. Aneuploidy in Batrachochytrium Dendrobatidis

Although most fungi are considered to have zygotic meiosis where the diploid stage is highly limited, this is a gross generalization. In fact, there are no extensive studies of the genetics of Chytridiomycota that would allow ploidy changes during the life cycle to be fully elucidated. In *Bd*, an outcome of hybridization is the creation of genomes that are approximately 0.233% heterozygous [97]. This level of divergence is considerably lower than other hybrids, like the cryptococcal hybrids. and presumably should not provoke chromosome incompatibilities. Nonetheless, the presence of chromosomal rearrangements across *Bd* lineages has not been explored. Like *Cryptococcus*, *Bd* is also capable of generating genetic diversity via dynamic genome mutations including aneuploidy and LOH. While the *Bd* genome is generally considered to be diploid, chromosomal copy number can vary greatly. Whole genome sequencing studies of *Bd* finds a high variation in aneuploidy within individuals and among closely related isolates. Chromosomal copy number in *Bd* can vary between 1 (monosomic) to 5 (pentasomic). In 51 *Bd* genomes that have been sequenced and analyzed for copy number variation, two studies together show that approximately 58.7% of *Bd* chromosomes are disomic, 29.0% are trisomic, 11.3% are tetrasomic, and less than 1% monosomic or pentasomic [97,103].

The widespread nature of aneuploidy across major lineages of *Bd* suggests that it may be an important mechanism in the generation of genetic variation, especially given the rarity of sexual reproduction observed in this species. Links between variation in chromosomal copy number and phenotypic effect, however, have been difficult to establish in this species. This is largely due to the paucity of phenotyped *Bd* isolates with accompanying whole genome sequences. Comparatively, no obvious patterns have emerged to link highly aneuploid genomes to the hypervirulent BdGPL clade. The BdGPL lineage contains representative isolates displaying disomic, trisomic, and tetrasomic genomes throughout its phylogeny [97,103]. While the sample sizes of analyzed genomes outside the better-studied BdGPL clade are very small, some phylogenetic trends are beginning to emerge around *Bd* aneuploidy with respect to lineage. In the novel, enzootic *Bd* lineages, the BdCape isolates analyzed so far (*n* = 5) show a mix of trisomic (60%) and tetrasomic (40%) genomes [103]. Three representative isolates assigned to BdCH were mostly trisomic. BdBrazil isolates (*n* = 2) were mostly disomic [97]. Finally, the single BdGPL/BdBrazil hybrid analyzed is also mostly disomic, with 13/17 of its major chromosomes disomic and 4/17 trisomic [97]. 

In addition to observations of widespread aneuploidy in *Bd*, chromosomal copy number has also been shown to be mutable over short timescales. Farrer et al. [103] examined replicate laboratory lines of an ancestrally trisomic *Bd* isolate serially passaged over 40 passages under differing growth conditions. One line was passaged in a standard media while the other was passaged in a selective media containing defensive antimicrobial peptides collected from the European water frog (*Pelophylax esculentus*) [104]. After approximately 40 weekly passages, the culture sequenced from standard media lost a copy of one chromosome (supercontig IV) and gained a copy of another (supercontig V), while the culture passaged in antimicrobial peptide media gained a chromosomal copy at supercontig V. Another study investigated the genomic changes in an isolate, JEL427, before and after 30 transfers in the lab [105]. The isolate showed lower virulence and spore production after the passages [106]. The major change in the isolate over the 30 passages was a reduction in ploidy, going from an average of 3.6 copies per chromosome (in practice genomic scaffold as the genomic map is not available) to 3.1. The difference between the two laboratory evolution studies may relate to their divergent starting points, but both results show that aneuploidy changes can occur rapidly in *Bd*, perhaps serving as a mechanism for rapid genomic adaptation to changing selective pressures. While the functional underpinnings of these pattern are difficult to tease out, they may suggest that specific *Bd* chromosomes more readily gain or lose copies. This pattern is also reported in another major study investigating aneuploidy in *Bd*, where the authors find that supercontig V is one of the *Bd* chromosomes more likely to present higher than average copy numbers [97].

Characterization of the ploidy of additional hybrid isolates will be essential for understanding whether meiosis and sex result from the fusion of haploid gametes or through a parasexual cycle. The general reduction in chromosome number over time from an ancestor of higher than disomic average chromosome numbers would tend to support parasexuality, however, the absence of more than two alleles per locus suggests that it is more likely that an endoduplication of all or part of the genome occurs sometime after hybridization via a heretofore undetected standard sexual cycle. Either way, hybrid genotypes present a greater deal of allelic diversity, which could facilitate adaptation by LOH. Understanding whether LOH occurs primarily at the chromosome level, i.e., aneuploidy, or at the gene level, e.g., gene conversion, in hybrid isolates is critical for understanding the nature and magnitude of the selective forces they experience.

### 5.5. LOH in Batrachochytrium Dendrobatidis

Loss of heterozygosity is a well-known feature contributing to genome diversity in *Bd*. LOH is hypothesized to occur during asexual reproduction of *Bd* through mitotic recombination, chromosome loss, or gene conversion [96]. In addition to changes in chromosome copy number, LOH has a great capacity to generate genotypic diversity without the input of new alleles [107]. This may be particularly important in clonally dominant pathogen lineages such as in *Bd*, such as the BdGPL lineage which typically shows a maximum of two alleles per locus, despite being frequently trisomic. The genotypic diversity generated by LOH should alter combinations of alleles within and across loci, displaying overdominance, underdominance, or epistasis, which can then be subject to selection pressure with presumably advantageous tracts of LOH sweeping to fixation in a population. 

The most prominent example of this in *Bd* may be a large, shared LOH region on supercontig II that is present in all members of BdGPL [92,97,103]. The conserved nature of this LOH feature throughout the globally invasive clade may reveal clues to the successful proliferation of this lineage. Gene functions, enriched in shared LOH regions of BdGPL, included processes related to reactive oxygen metabolism, L-serine metabolism, and superoxide dismutase/oxidoreductase [97]. These gene classes, in addition to various peptidases identified through comparative genomics with the closely related non-pathogenic chytrid *Homolaphlyctis polyrhiza* [108], and transcriptomic studies of *Bd* infections [109,110], are possibly involved in the genomic evolution underpinning the adaptive success of BdGPL in varied habitats worldwide.

In clonally reproducing diploid organisms such as *Bd*, the lack of outcrossing results in regions of LOH in the genome persisting in lineages through time. Because of this, shared homologous regions of LOH can be a powerful tool to inform populations of closely related clonal strains. For example, homologies in patterns of LOH have been used to distinguish evolutionary subclades within the global BdGPL [96,107]. Further geographic sampling and improved computational methods to detect homologies in LOH patterns hold the promise of resolving their finer-scale, intra-lineage population structure, and providing a more refined picture of the geographic history of this ecologically important pathogen.

## 6. Conclusions and Perspectives

Hybridization and genomic plasticity appear to be shared hallmarks contributing to rapid adaptation in fungal pathogens. The shared mechanisms of hybridization, aneuploidy, and LOH between the two major fungal pathogens *Cryptococcus* and *B. dendrobatidis* span the fungal tree of life (phylum Basidiomycota to Chytridiomycota) and appear to reflect a global pathway for rapid adaptation in pathogens across the fungal kingdom. Themes emerging from multiple studies of fungal hybrids are that they demonstrate increased ploidy, heterosis, and novel ecological niches. Prime examples of successful hybrids with these traits across the fungal tree of life are readily found. For example, hybridization in the plant endophytic species *Epichlöe* leads to asexual diploids/polyploids with major benefits to the host, and hence the fungus [111]. Likewise for the pathogen, *Verticillium longisporum*, hybridization is associated with an expanded host range and diploidization [112,113]. Ancient hybridization is involved in the diversification of Saccharomycete yeasts [114], while more recent hybridization and polyploidization is involved in the formation of yeasts (*Saccharomyces* spp.) involved in beer brewing [115,116]. Finally, two examples of human pathogenic species appear to be largely of hybrid origin: the halophilic black yeast *Hortaea warnecki* is the cause of superficial skin infection, tinea nigra [117] and the recently described, but rare, *Candida metapsilosis* [118]. 

The above-mentioned fungal hybrids generally are discovered as F1 and have higher ploidy than their parental species or lineages. This appears to be the case for *Cryptococcus* hybrids but not for *Bd*, for which hybrids are the same ploidy as the parental genotypes. *Bd* hybrids are therefore similar to homoploid hybrids, good examples of which, in fungi, are *Zymoseptoria pseudotritici* and *Microbotryum* spp. [119,120]. Following the formation of F1 hybrids, persistence and adaptation is facilitated by the mechanisms of genomic plasticity during asexual growth, such as ploidy cycling and LOH. The high number of successful asexual F1 hybrids with heterosis across species suggests that any reproductive isolation caused by genetic incompatibilities are likely to be recessive as predicted by theory [1]. Overall, however, it is plausible that the importance of hybridization in fungal adaptation may be overinterpreted, as the likelihood of identifying or observing failed hybrids is low. The community would benefit from additional work synthesizing hybrids in the lab to understand and predict the outcome of hybridization on evolution, such as has been conducted for investigating reproductive isolation in *Neurospora* and *Microbotryum* [121,122]. More needs to be known about fungal adaptation to ecological gradients in order to understand whether hybrid zones are likely to exist at the boundaries in which allopatric species meet.

Understanding the evolutionary dynamics of better studied pathogens such as *Cryptococcus* can inform the biology of less characterized, or newly discovered, fungal pathogens such as *Bd*. As we have discussed, the *Cryptococcus* system has served as a model for establishing the relationship between divergence, chromosomal pairing, and sterility. As *Bd* hybrids are not as diverged as *Cryptococcus*, and may not even represent the fusion of distinct species, we predict that F2 and further generation of hybrids of *Bd* are likely to exist, and it would be appropriate to account for this possibility when conducting additional sampling in regions of admixture, perhaps even as more subtle forms of introgression. Model species, like *Cryptococcus*, not only allow specific gene hypotheses to be tested through gene manipulation [123], they also generally have easy to trigger sexual cycles and selectable markers that allow for crossing designs to understand the genetics of complex traits [66]. As another example, crosses have established that the mitochondrial genotype in *Cryptococcus* is not known to have an impact on hybrid fitness, yet this has been demonstrated in other species of fungal hybrids, using crossing designs that create identical nuclear genomes in alternative mitochondrial association [79,124]. More work should be done to develop a crossing system in *Bd* that could allow for mitochondrial–nuclear genetic interactions to be tested. Finally, in *Cryptococcus*, same-mating-type mating was first demonstrated to occur in nature, because the mating type locus had been well described and easily genotyped [37]. This is a large hurdle for a species like *Bd*, but the fact that *Bd* hybrids exist should provide impetus to determine the genetic basis of mating types which may reveal similar same-mating-type mating dynamics.

Regardless of the underlying genetic differences between *Cryptococcus* and *Bd*, this review has highlighted several commonalities regarding their hybridization. Hybrids in both genera show increased genomic plasticity, with the potential to generate transgressive phenotypes, unleashed following both meiotic and mitotic recombination. Hybrids in both genera reveal that it is a subset of environments, rather than all, in which they have higher fitness. Finally, the spread of both pathogen genera likely involves human-assisted migration that led to subsequent admixture. Given the potential adaptive benefits of hybridization in both *Cryptococcus* and *Bd*, improved measures for pathogen containment to prevent increased opportunities for hybridization should be put in place, both in clinical and environmental settings.
